# Managing the Microbial Community of Marine Fish Larvae: A Holistic Perspective for Larviculture

**DOI:** 10.3389/fmicb.2018.01820

**Published:** 2018-08-27

**Authors:** Olav Vadstein, Kari J. K. Attramadal, Ingrid Bakke, Torunn Forberg, Yngvar Olsen, Marc Verdegem, Cristos Giatsis, Jorunn Skjermo, Inga M. Aasen, François-Joel Gatesoupe, Kristof Dierckens, Patrick Sorgeloos, Peter Bossier

**Affiliations:** ^1^Department of Biotechnology and Food Science, Norwegian University of Science and Technology, Trondheim, Norway; ^2^Department of Biology, NTNU Norwegian University of Science and Technology, Trondheim, Norway; ^3^Aquaculture and Fisheries Group, Wageningen University, Wageningen, Netherlands; ^4^Department of Environment and New Resources, SINTEF Ocean, Trondheim, Norway; ^5^Department of Biotechnology and Nanomedicine, SINTEF Industry, Trondheim, Norway; ^6^UMR 1419 NuMeA, INRA, Université de Pau et des Pays de l'Adour, Paris, France; ^7^Faculty of Bioscience Engineering, Laboratory of Aquaculture and Artemia Reference Center, Ghent University, Ghent, Belgium

**Keywords:** aquaculture, microbial management, microbe-host interactions, microbe-microbe interaction, aquaculture systems, bacterial flows

## Abstract

The availability of high-quality juveniles is a bottleneck in the farming of many marine fish species. Detrimental larvae-microbe interactions are a main reason for poor viability and quality in larval rearing. In this review, we explore the microbial community of fish larvae from an ecological and eco-physiological perspective, with the aim to develop the knowledge basis for microbial management. The larvae are exposed to a huge number of microbes from external and internal sources in intensive aquaculture, but their relative importance depend on the rearing technology used (especially flow-through vs. recirculating systems) and the retention time of the water in the fish tanks. Generally, focus has been on microbes entering the system, but microbes from growth within the system is normally a substantial part of the microbes encountered by larvae. Culture independent methods have revealed an unexpected high richness of bacterial species associated with larvae, with 100–250 operational taxonomic units associated with one individual. The microbiota of larvae changes rapidly until metamorphosis, most likely due to changes in the selection pressure in the digestive tract caused by changes in host-microbe and microbe-microbe interactions. Even though the microbiota of larvae is distinctly different from the microbiota of the water and the live food, the microbiota of the water strongly affects the microbiota of the larvae. We are in the early phase of understanding larvae-microbe interactions *in vivo*, but some studies with other animals than fish emphasize that we so far have underestimated the complexity of these interactions. We present examples demonstrating the diversity of these interactions. A large variety of microbial management methods exist, focusing on non-selective reduction of microbes, selective enhancement of microbes, and on improvement of the resistance of larvae against microbes. However, relatively few methods have been studied extensively. We believe that there is a lot to gain by increasing the diversity of approaches for microbial management. As many microbial management methods are perturbations of the microbial community, we argue that ecological theory is needed to foresee and test for longer term consequences in microbe-microbe and microbe-larvae interactions. We finally make some recommendations for future research and development.

## Introduction

**Figure 1:**
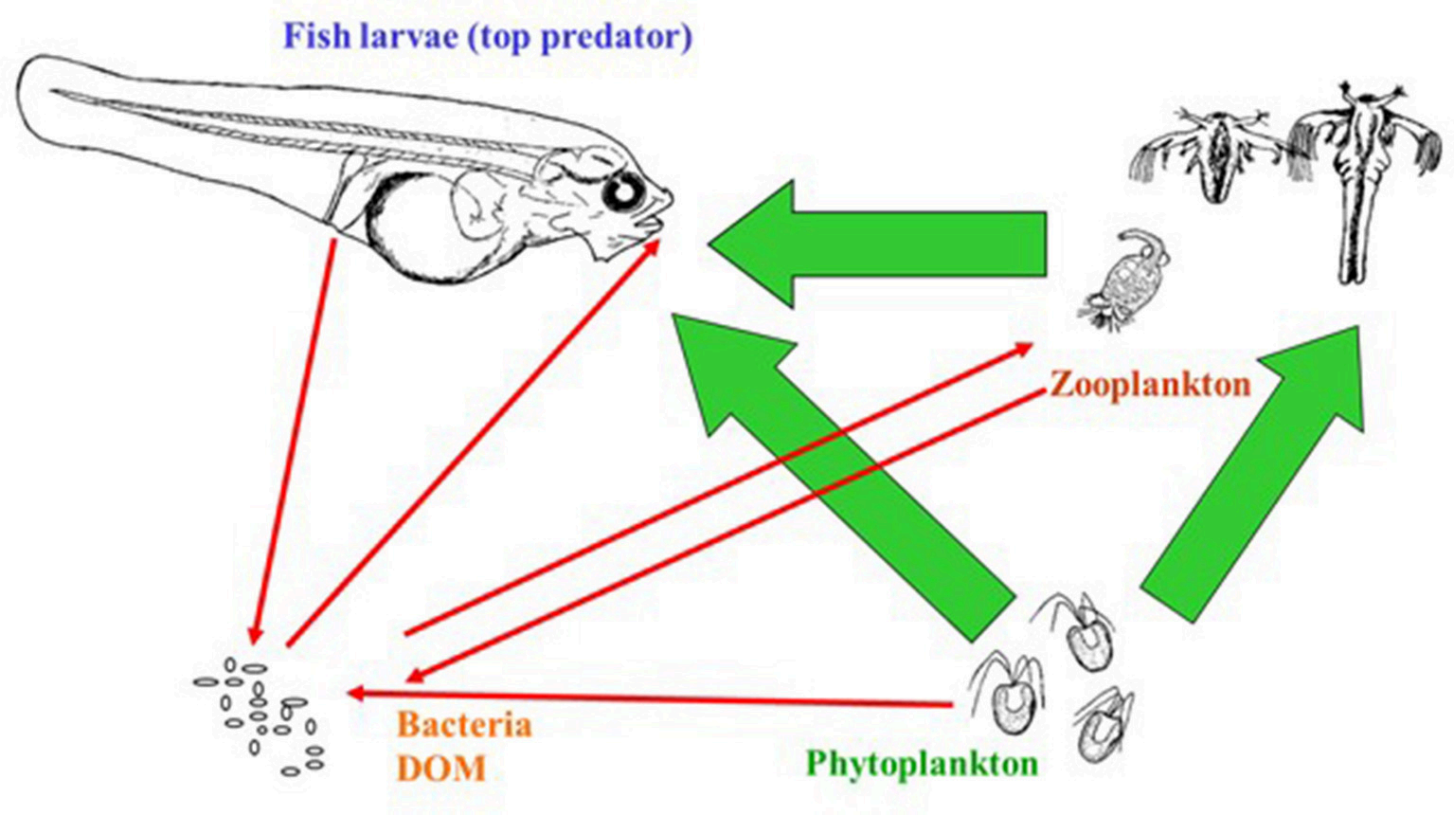
First feeding of larvae in a complex food web. Arrows indicate direction of effects in the direction of the arrow. Interactions between dissolved organic matter (DOM) and bacteria, and bacteria-bacteria interactions are not indicated. In addition to interactions ongoing within the rearing tank, various inputs and losses from the tank also affect the microbe-dominated food web.

The main object in aquaculture is of course high survival and quality of the reared species. For marine fish larvae live zooplankton prey has to be offered because the survival and performance of the larvae is poor on formulated diets. Furthermore, it is common practice to add live or inactivated microalgae to the rearing systems as this has shown to be beneficial for the larvae. The community of the rearing tanks consist accordingly of three biological compartments with three known interactions (predation) between them (Figure 1, green arrows). However, when bacteria and dissolved organic matter (DOM) serving as growth substrate for the heterotrophic bacteria are included in this picture, the simple food web with three interactions becomes a complicated food web with a large number of interactions. In Figure 1 five red interaction arrows are illustrated, but this represents a simplification as most arrows to the Bacteria/DOM pool are supplying a mixture of both bacteria and DOM, and DOM consumption is done by a large number of bacterial populations (>100; e.g., Giatsis et al., 2014; Bakke et al., 2015). Thus, the first feeding rearing tank is a complex ecosystem with likely several hundred interactions. Despite a growing acceptance of detrimental larvae-microbe interactions as a key reason for the poor performance during larval rearing (e.g., Vadstein et al., 1993a, 2013; Munro et al., 1995), we have a rudimentary comprehension of the intricate web of interactions the microbes are involved in.

The cultivation system has implications for the microbial communities in the tanks, and is therefore briefly discussed below. The traditional way to rear fish in tanks is flow-through systems (FTS), with a continuous flow-through of water. The intake water in a FTS is normally treated to meet the requirements of the cultured species in its different life stages, and to protect the larvae from pathogenic organisms. Common treatment practices and techniques used for intake water in a FTS are particle removal, temperature regulation, aeration/degassing and disinfection (Lekang, 2007). With respect to the microbes the treatments aim to establish a microbial barrier between the outside and the rearing facility, and for a general reduction of densities of microorganisms. However, the microbial quality of the water that leaves the initial treatment system is not identical to the microbial quality experienced by the cultured organism in the rearing tanks, as the time delay between treatment and water leaving the rearing tank allows for re-colonization (Vadstein et al., 2004; Attramadal et al., 2014). In FTS this is an uncontrolled process which selects for fast-growing r-strategic opportunistic bacteria (see later) due to high substrate availability per microbe. Such bacteria were 25 years ago hypothesized to be detrimental to fish (Vadstein et al., 1993a), and has since then been supported by several studies (reviewed by Vadstein et al., in press). The symptoms on the fish larvae due to exposure to a microbial community dominated by r-strategist bacteria can be summarized as poor performance and lack of reproducibility, and is due to their ability to over-colonize stressed hosts and create detrimental fish-microbe interactions (Vadstein et al., in press).

To overcome the problems associated with r-selected communities, a controlled re-colonization under K-selection in a biofilter—termed microbially matured system (MMS)–was proposed by Vadstein et al. (1993a). In MMS the water is first disinfected, but then the water is re-colonization in a controlled manner in a header tank with a biofilter (Skjermo et al., 1997) before the water is going to larval rearing tanks. The high microbial biomass in the maturation unit, mainly attached to the biofilter, results in low substrate availability per microbe and hence K-selection for competition specialists. K-strategists are non-opportunistic, suppress r-strategists, and are hypothesized to promote healthy host-microbe interactions (Vadstein et al., 1993a). A considerable number of studies have supported this hypothesis (reviewed by Vadstein et al., in press). In MMS the densities of microbes are comparable to those in FTS, but the composition of the microbial community is different (see below). However, the density of microbes in the incoming water to rearing tanks in FTS and MMS is considerably lower than the microbial density in the rearing tanks due to feeding.

In addition to the two flow-through systems, recirculating aquaculture systems (RAS) with a continuous restoration and reuse of the water is used for cultivating aquatic organisms (Blancheton et al., 2013). In RAS the water is treated to remove inorganic and organic waste, and to replace oxygen lost due to metabolism, and targets reduction of particles, DOM, ammonia, nitrite, and carbon dioxide produced by all organisms in the rearing tanks (cultivated organism, live food, and microbes). The motivation for RAS has been to save energy and water, and the ease of waste handling. Recently it has been proposed that RAS also secures K-selection, and RAS should therefore also be considered a microbial management strategy (Attramadal et al., 2012c). Water entering the cultivation tanks in a RAS system has microbial densities comparable to the densities in the cultivation tanks (Attramadal et al., 2012c). The composition of the microbial communities are described and discuss in more detail below.

It has been assumed that the microbiota of newly hatched larvae largely is established in a non-selective manner (see also Figure 1), but host preference, competitive ability (Makridis et al., 2000b; Vadstein et al., 2004; Bakke et al., 2015) and neural processes (i.e., drift and dispersal) (Burns et al., 2016) are also important. Most previous studies on characterization of the microbiota have used samples with pooling of individuals and culture dependent approaches. However, a recent study on Atlantic cod found variation in the composition of the microbiota among individual larvae within one tank comparable to the variation between rearing facilities with different holding regime (Fjellheim et al., 2012). Similar observations have been made with other animals (Simpson et al., 2000; Zhu et al., 2002; Suchodolski et al., 2004; Turnbaugh et al., 2009). It still needs to be verified whether this individual variation is characteristic for fish larvae, but more importantly to what extent this broad variation in microbiota of larvae has biological implications for the host. It has been shown for humans that whereas the composition of the microbiota vary considerably between individuals, the metagenome of the microbiota is remarkably similar (Turnbaugh et al., 2009). Thus, the functions needed in an ecosystem can be promoted by different species inventory, and not a specific species composition.

Moreover, Fjellheim et al. (2012) revealed poor correlation between the culture dependent and the culture independent approach in terms of both quantity of microbes and in diversity. It is known that cultivation-based methods give both underestimation of densities (Amann et al., 1995) and bias in community composition (Hugenholtz et al., 1998), and these data have limited credibility today. The basic questions, i.e., which microorganisms are colonizing developing larvae and which function do they have, still needs to be addressed in a proper way. The fast developing high throughput DNA sequencing technologies have come to the rescue, by generating a vast amount of data (Bakke et al., 2015; Vestrum et al., 2018). The next step will be to use these new techniques to get more knowledge on what the microbiota do.

Knowing which microorganisms that colonize larvae is baseline information. It is more important to investigate their *in situ* activity and how that activity influences the host. The mechanisms by which bacteria interact positively and negatively with fish larvae are not understood (Tinh et al., 2008). The benefits to the host from a healthy intestinal microbiota is well described for ruminants and hypothesized for other groups of animals. However, relatively little is known *de facto* for fish (Ringø and Birkbeck, 1999; Gatesoupe, 2008). Moreover, the tools required to study these mechanisms have until recently been inadequate. Possible hypotheses for the beneficial effects of a healthy microbiota on the host include: competition and antagonism vs. detrimental bacteria, stimulation of the immune system, provision of nutritional factors, including digestive enzymes and vitamins, and transcriptional effects at the host level including cell differentiation, metabolisms and stimulation of the immune system.

A clarification of the community level role of the microbiota for host functionality will provide fundamental information with large practical implications for the aquaculture industry. Hence, we need to improve our understanding of these interactions to benefit viability and robustness of the fish in aquaculture. This “join them” approach considering the microbial community is contradictory to the traditional “beat them” strategy generally applied in microbial management in medicine, agriculture and aquaculture, with an exception for use of probiotics (De Schryver and Vadstein, 2014). Considering the fact that detailed information on microbial activity in larval stages is scarce, this review will present some concepts that need to be substantiated in the future.

This review look at the microbial community of fish larvae from an ecological and eco-physiological perspective. Firstly, this is a number game; i.e., how many microorganisms from different sources reach the larvae under different rearing regimes. Secondly, we summarize the current knowledge on the microbial species that colonize larvae, and try to assess *in vivo* microbial activity that is beneficial or detrimental to the host. Thirdly, we discuss status and prospect of various microbial management methods that can be used in aquaculture. Finally, we make recommendations for future research and development. We conclude that there is need for a change of paradigm—the total microbial community should be considered in the development of microbial management strategies and the microbial community should be used as positive actor for a more sustainable aquaculture.

## Bacterial flow in larval tanks and bacterial load to larvae

### General concept and premises

**Figure 2:**
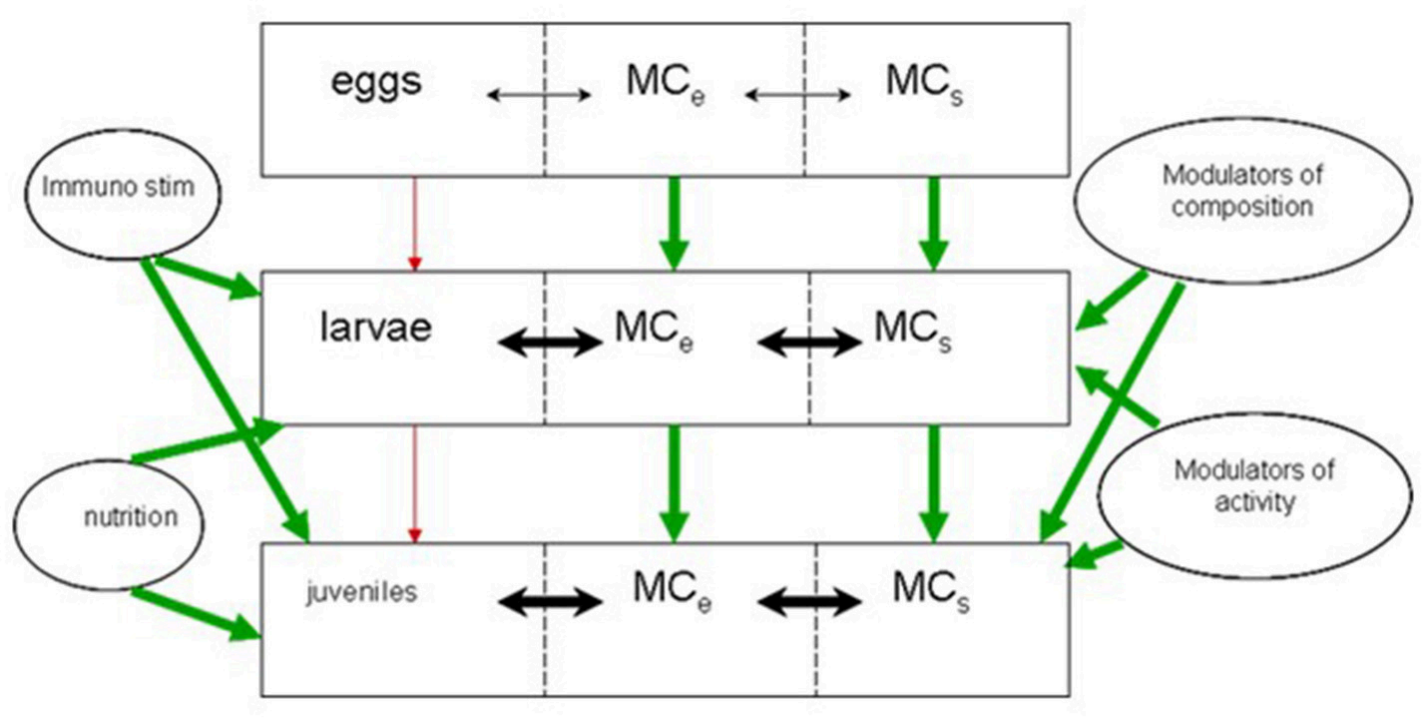
Conceptual framework for the microbial interaction between system and host MC_e_: Microbial community of biological components, MC_s_: Microbial community of the system, water and non-living components.

The larvae are continuously exposed to flows of bacteria of different origin (MC_S_, Figures 1, 2), and these flows are dependent on water flow rates and bacterial densities in the process water. Bacterial densities in rearing tanks are affected by bacterial densities in process water, bacteria added with live food and microalgae, and bacterial growth in the larval tanks. The bacterial load associated with the live food components (microalgae, rotifers, and *Artemia*) is variable, dependent of the cultivation methods, and on the washing and disinfection procedures of the live food cultures. Moreover, treatment of the water supply to larval tanks will affect bacterial numbers in the inflowing water and in the water of the larval tanks, or in MC_S_. Finally, the internal processes of bacterial growth are dependent on the rearing system and the overall supply of organic substrates.

Below the bacterial flows in larval tanks are quantified for three different aquaculture systems characterized by different bacterial densities and dynamics: (1) *Flow through systems* (FTS): Intake water is filtered and disinfected. (2) *Flow through maturated systems* (MMS): Intake water is filtered and disinfected, and thereafter recolonized by bacteria by allowing >8 h retention time in an aerated biofilter (Skjermo et al., 1997; Salvesen et al., 1999). (3) *Recirculating aquaculture systems* (RAS): Water is initially treated like FTS, but the used water is passed through a biological filter and other water treatment components (e.g., filters and protein skimmer), and reused several times.

Beside the bacteria in the rearing water, other bacterial sources for larvae tanks are:

- Bacteria in cultures of microalgae
- Bacteria associated with live food (rotifers, copepods, and *Artemia*).
- Bacteria produced in larval tanks.

The exposure mechanism of bacteria from live food and microalgae to the larvae is different from that of bacteria in the process water, including both input and bacterial growth within the system. If the supply of bacteria to the rearing tank were only from process water the bacterial density would be constant with time, and only dependent on the abundance of bacteria in the inflowing water. The microbial density in the larval tanks would in this case not be reduced through the process of water exchange. The input of bacteria from live food or through internal growth, will increase the density of bacteria in the rearing tank, and depends on the magnitude of the supply and the exchange rate of water of the larval tanks. The importance of supply from food and internal growth are more important when the dilution rate of the tanks is low. For very high dilution rates, the importance of internal bacterial growth is expected to be low and increasingly dependent on growth by bacteria attached to surfaces of the system. The bacteria from live food, and microalgae are continuously and rapidly diluted between each feeding.

Beside these quantitative aspects, there will also be a change in the composition of the microbial community associated to live food, microalgae, and water (see below). The half-time of this exchange process appears to be hours to a day, and therefore of approximately the same timescale as the retention time of the water in larval tanks. This affects the bacterial composition consumed by the larvae. The density of other bacterial populations supplied to the larval tanks, e.g., probiotic bacteria, will also depend on the dilution rate of the tank and the ability of these bacteria to grow or become associated to live food and surfaces in the system.

### Input data for estimation of bacterial flows

Table 1 reviews typical bacterial numbers in the different rearing systems and in the live food. The numbers of bacteria are highest in RAS-water, lowest in MMS-water and intermediate in FTS-water. These differences among systems, ranging up to one order of magnitude, are substantial and believed to be representative. The absolute number of bacteria can be affected by the water source.

**Table 1:** Bacterial densities of main sources during larval rearing in three different types of rearing systems.

**Aquaculture system**	**Inlet water**	**References**
FTS	0.2 × 10^6^ mL^−1^	Attramadal et al., 2014
MMS	0.1 × 10^6^ mL^−1^	Attramadal et al., 2014
RAS	2.5 × 10^6^ mL^−1^	Attramadal et al., 2014
**Live feed/algae**	**Live food added**	
Rotifers	10^4^ per rotifer	Skjermo and Vadstein, 1993
Artemia	5 × 10^4^ per *Artemia*	Olsen et al., 2000
Microalgae culture	4 per algal cell	Salvesen et al., 2000

*FTS, Flow-through System, MMS, Microbially Matured System, and RAS, Recirculating Aquaculture System*.

The present analysis of bacterial flows, including (1) to and in rearing tanks and (2) into the larvae, are made for rearing conditions and a feeding regime representative for Atlantic cod larvae. The quantitative analysis of bacterial flows of larval tanks from 3 to 30 days post hatching (dph) was made for the following rearing conditions based on Attramadal et al. (2014):

- The age-dependent variable dilution rate of tank water is 2, 3, 5, and 6 day^−1^ for larval stages of 3, 10, 19, and 30 dph, respectively
- Rotifers are added daily from 2 till 17 dph
- *Artemia* are added daily from 18 to 30 dph

Input variables for the bacterial densities in different sources used in the analysis are shown in Table 1. The numbers of rotifers and *Artemia* consumed per larva was calculated from the amount of food needed to sustain optimal growth and survival of cod larvae, using an existing spreadsheet model simulating survival, consumption of food, and daily larval growth increment from 3 to 30 dph. A growth yield of 20%, was assumed for the fish larvae. The simulations were made for an initial stocking density of 100 larvae L^−1^ and a survival of 30% at 30 dph. The estimated drinking rate (DR) of larvae was based on data for cod (Mangor-Jensen and Adoff, 1987; Tytler and Blaxter, 1988), and an exponential function fitted to the data was used to calculate DR. Bacterial clearance rate by larvae was calculated as 100·DR (Reitan et al., 1998), and predation of bacteria equal clearance rate multiplied by bacterial density. Bacterial growth in fish tanks was measured as the incorporation of ^3^H-leucine into bacterial protein (Attramadal et al., 2014). Typically, algae are added from start and along with the rotifers (until 17 dph). Bacterial densities in algal cultures vary greatly with species and cultivation condition (Salvesen et al., 2000). We assume that addition of 1 mg C L^−1^ of *Isochrysis galbana* for “green water” at the start (fill up), contribute 2.2·10^8^ CFU L^−1^ day^−1^ to the fish tanks, and 16–100% of the bacteria are culturable (Reitan et al., 1993; Salvesen et al., 2000). In the presented analysis, we estimate that addition of algae contributed with 5·10^8^ bacteria L^−1^ day^−1^ to the rearing tanks (one single addition).

**Figure 3:**
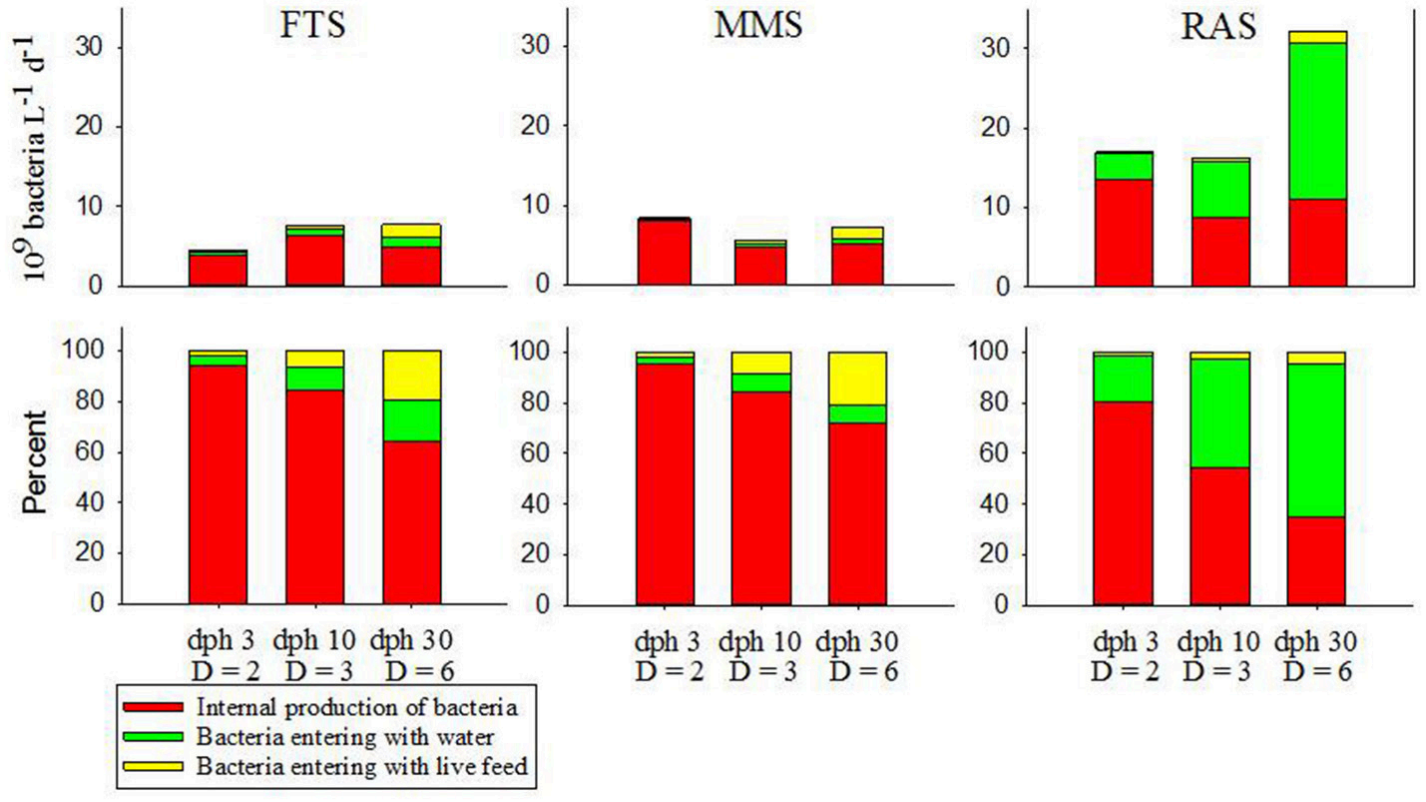
Flows of bacteria to larval tanks per day from the principle external sources and the internal growth of bacteria in the rearing tanks in three water management systems for selected days post hatch (dph) of Atlantic cod. Different stages during the first feeding period have different tank dilution rates (D, tank volumes exchanged d^−1^) and different live food (3 and 10 dph with rotifers and 29 dph with *Artemia*). Top panels show absolute numbers and bottom panels show percent.

In Table 2 we have included addition of algae to illustrate the influence from this source of bacteria on the rearing water of the different systems. Addition of microalgae is an important contributor of bacteria during the rotifer period, especially when the exchange rate of tank water is low. However, because of the large uncertainty in the number of bacteria brought in with each volume of algal culture added to the tanks and that for adjusting contrast in the rearing tanks algae paste and clay are often used instead of microalgae (Attramadal et al., 2012b), we do not include this sources of bacteria after start-up (Figure 3).

**Table 2:** First day supply of bacteria following addition of water and live food for an initial stocking density of 100 larvae L^−1^.

**Bacterial source**	**Loading rate (× 10^6^ L^−1^ day^−1^)**
Inflowing water/system (filling of tanks at start)	200 (MMS) 500 (FTS) 2,000 (RAS)
Rotifers, addition of 5000 rotifers L^−1^	50
Microalgae, addition of 1.3 × 10^8^ algae cells L^−1^	500
New bacterial biomass production in larval tanks	8,000

### Sources of bacteria to and in larval tanks with cod larvae

The supply of bacteria from the water and the first addition of live feed and microalgae, creates an initial situation shown in Table 2. In RAS, the water is the dominant source of bacteria in the initial situation. For FTS the water and addition of algae contributes at the same level, whereas in MMS the water is less dominant. Thus, addition of microalgae becomes relatively important for MMS and FTS, whereas bacteria supplied with the live food is not that important. Microalgae can be more important in all systems if tanks are kept stagnant for some days.

Bacterial growth in the rearing tanks (Table 2; Attramadal et al., 2014) can be significant, especially at low dilution rates. If the hatching takes place in the first-feeding tanks, and not in separate systems, the larval tanks will get a significant pulse of organic substrates following hatching (ca 20–25% of total egg carbon for turbot, unpublished data). This pulse is proportional to the stocking density, and it may boost internal bacterial growth of opportunistic bacteria if the stocking density is high. Hatching in first-feeding tanks should thus be avoided.

Figure 3 shows the estimated flows of bacteria to larval tanks and production of bacteria in tanks for the three water treatment systems for selected time points during first feeding. The upper panels show the quantitative rates and the lower panels the percent contribution from the different flows. The bacterial supply rates in all systems are dominated by the production of bacteria in the tanks from the start. However, RAS is the only system with a significant contribution from another source, i.e., bacteria in the incoming water. There is a gradual increase in the influence of the microbial community of the process water from MMS to FTS and to RAS. The quantitative contribution of the live food on bacteria in the rearing water is relatively low in all systems, but it increases with larval age and is highest during feeding with *Artemia* at 30 dph. This is particularly the case in MMS where the bacterial density of the process water is the lowest.

It is notable that in RAS the importance of the microbial community of the process water is similar to the internal production of bacteria in the larval tanks, and supply from the process water become the dominant source of bacteria to RAS-tanks during the *Artemia* period. This indicates that the control or manipulation of the composition and number of bacteria in the inflowing (and initial fill-up) water can be a key to microbial control in the rearing tanks. It also suggests that the level of bacterial substrate (organic matter) in the rearing tanks likely control the internal growth of bacteria, and therefore the relative influence of the incoming water. If the water of the RAS is disinfected just before entering the rearing tanks, the system will look more like the FTS and the MMS because of the low number of bacteria entering the tanks.

The high influence of bacteria supplied with rotifers and microalgae in the early stagnant phase, represents another option of steering the microbial community of the rearing tank. Control can be achieved by careful preparation and manipulation of the bacteria of live food and microalgae, and by choosing a specific and well-functioning water treatment for the initial stage. See Olsen et al. (2000) for the effect of treating *Artemia*. It is also apparent that the effect of adding probiotic bacteria is strongest in the early phase of cultivation when dilution rates are low, and stagnant conditions may make such treatments even more efficient. The flow/supply analysis of bacteria in larval tanks is in agreement with the general concept depicted in Figure 2, and suggest no need for major revisions. A minor modification could be that different components of MC_s_ will interact differently with the host MC_e_.

**Figure 4:**
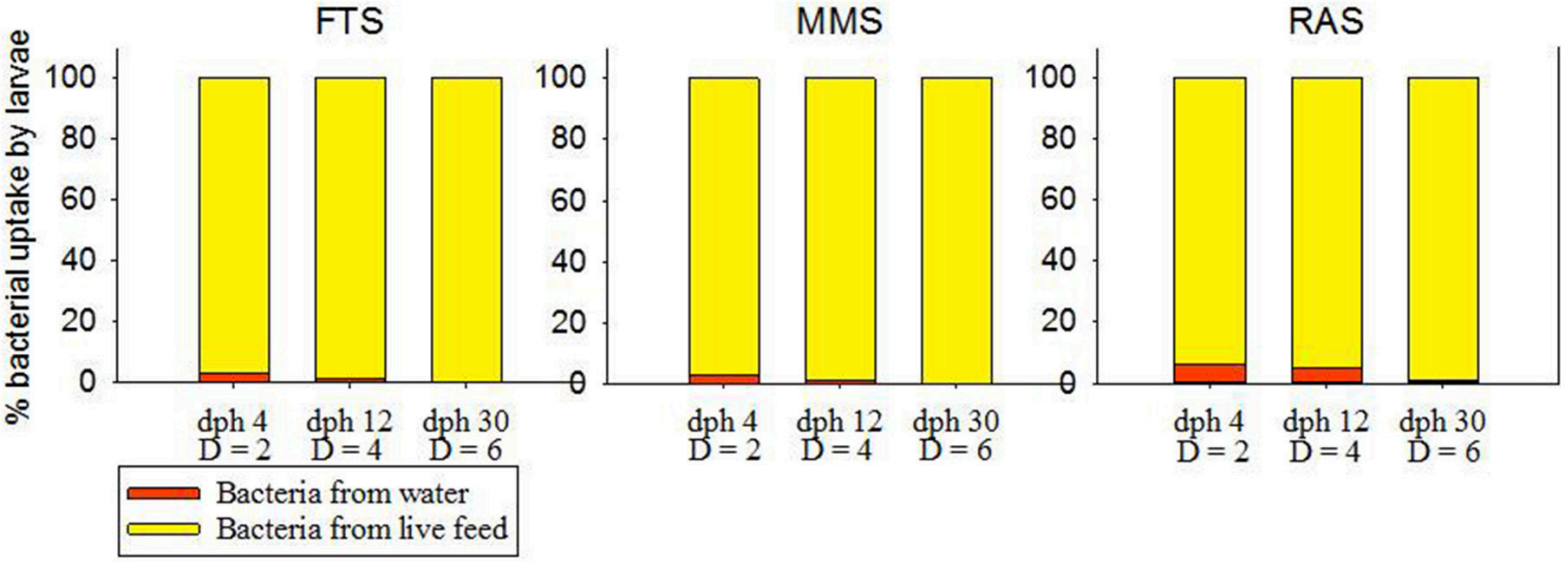
Fraction of the ingested bacteria per larva per day coming from the water and the live food, respectively, at different days post hatch (dph) characterized by different tank dilution rates (D, tank volumes exchanged d^−1^).

Figure 4 shows the relative influence of bacteria actively taken up from the water compared to the bacteria ingested with the live food. The average number of bacteria consumed per larva and day was estimated to 5·10^7^ in all three systems. At 4 dph the larval uptake of bacteria from the water was estimated to 2.9, 2.6 and 6.2% of the total uptake of bacteria from water and live food in FTS, MMS, and RAS, respectively. At 30 dph the uptake from the water is reduced to 0.1, 0.1 and 0.9%, respectively. The quantitative effect of the bacteria initially associated with the live food compared to the bacteria from the rearing water, will be reduced because of the exchange of bacteria between water and live food in the tank (Vadstein et al., 1993b; Makridis and Vadstein, 1999). However, some of the live food is consumed relatively fast and before its microbial community has been exchanged with bacteria in the water. In Figure 4 we have not included this effect, and we therefore show the maximum influence from the bacteria initially associated with the live food, and the minimum influence from the bacteria in the rearing water.

In conclusion, in all water treatment systems the uptake of bacteria by larvae is dominated by bacteria entering with the live food. However, the bacteria from the water also represent a significant contribution in all systems in the beginning of the live food period. In RAS the uptake of water bacteria by the larvae stays about the same throughout the first feeding phase, whereas it decreases in the other two systems with time. The RAS differs from the other two systems in the way that the water source is relatively more dominating, both on the rearing water community and on the larvae. This has implications for microbial management strategies.

## Community composition of microbiota associated with marine larvae

To obtain microbial control in aquaculture systems, we need to understand the processes governing the composition of the microbiota associated with different compartments; fish larvae, rearing water, algae, and live food. In addition, we need to know which processes that determine this composition. In several studies the microbiota associated with marine fish larvae and their rearing systems have been investigated by culture-based approaches or by amplified 16S rRNA gene fragment fingerprinting methods (Griffiths et al., 2001; Verner-Jeffreys et al., 2003; Jensen et al., 2004; Brunvold et al., 2007; McIntosh et al., 2008; Bjornsdottir et al., 2009; Sun et al., 2013). The taxonomic information obtained using such approaches is limited. Deep sequencing data based on 16S rDNA amplicons of teleost microbiomes are now accumulating (Llewellyn et al., 2014; Ghanbari et al., 2015), but the focus is on adult individuals. Deep sequencing give taxonomic results as Operational Taxonomic Units (OTUs), defined as all sequences with a DNA sequence similarity at a defined level (most common 97%). The taxonomic affiliation of an OTU is found by comparison with a reference database, and the resolution is normally on the genus level.

**Figure 5:**
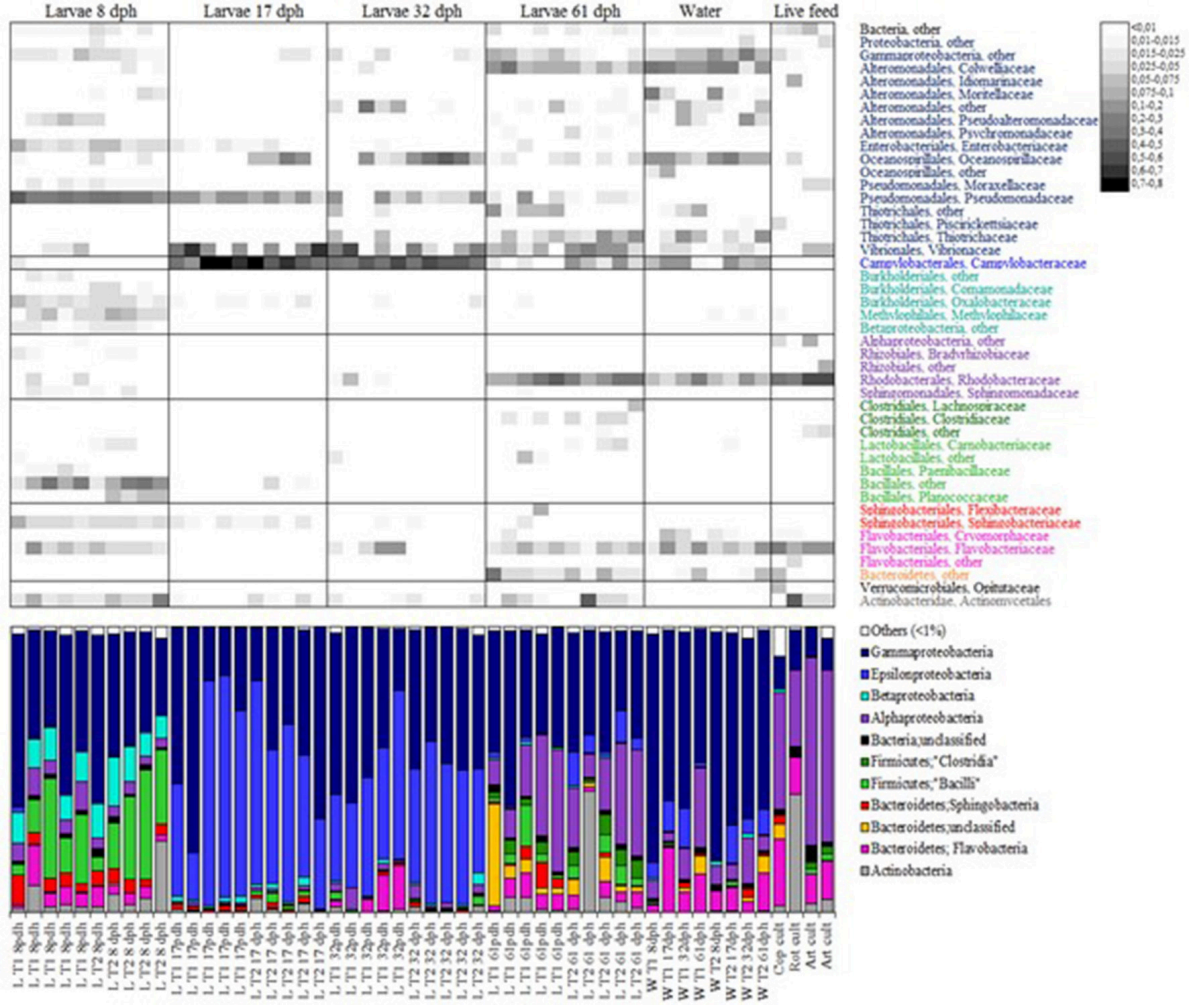
Relative abundance of bacterial phyla and families in individual cod larvae, water and live food samples determined by 16S rDNA amplicon sequencing. At the phylum level, the microbiota is presented as percent bar graphs (bottom), and at the family level as a heat map (top, scale upper right) with the abundance of each family represented by a colored block as specified in the figure. Bars labeled D8, D17, D32, and D61 represent cod larva individuals at the ages of 8, 17, 32, and 61 dph, respectively. Bars labeled W and F represent water and live food samples, and the time of sampling is indicated in the label. Only taxa represented by a proportion of ≥1% in at least one of the samples are shown (from Bakke et al., 2015). Reproduced with permission from John Wiley and Sons.

Recently microbiota associated with larvae of rainbow trout (Ingerslev et al., 2014), tilapia (Giatsis et al., 2015), and Atlantic cod (Bakke et al., 2015) has been examined by deep sequencing. To our knowledge, the latter is the only study providing detailed taxonomic information for a developing marine fish species for the composition of bacterial communities associated with developing cod larvae, rearing water and live food cultures. Individual fish larvae and water from two rearing tanks, representing different live food regimes (rotifers or copepods), were sampled at 8, 17, 32, and 61 dph. All cod larvae were fed *Artemia franciscana* from day 18 to 36 and formulated feed from 33 to 61 dph. Fifteen phyla were observed in the microbiota of cod larvae, with Proteobacteria, Firmicutes, Bacteroidetes, and Actinobacteria as the most abundant (Figure 5). In general, Proteobacteria, Firmicutes, and Bacteriodetes appear to be the dominating phyla in fish gut microbiota (Desai et al., 2012; Star et al., 2013; Ingerslev et al., 2014; Llewellyn et al., 2014). Representatives of the Acidobacteria, Chlamydiae, Cyanobacteria, Fusobacteria, Gemmatimonadetes, Nitrospira, Planctomycetes, Verrucomicrobia, and the candidate phyla TM7, OD1, and SR1, were sporadically present in the cod larval microbiota.

**Figure 6:**
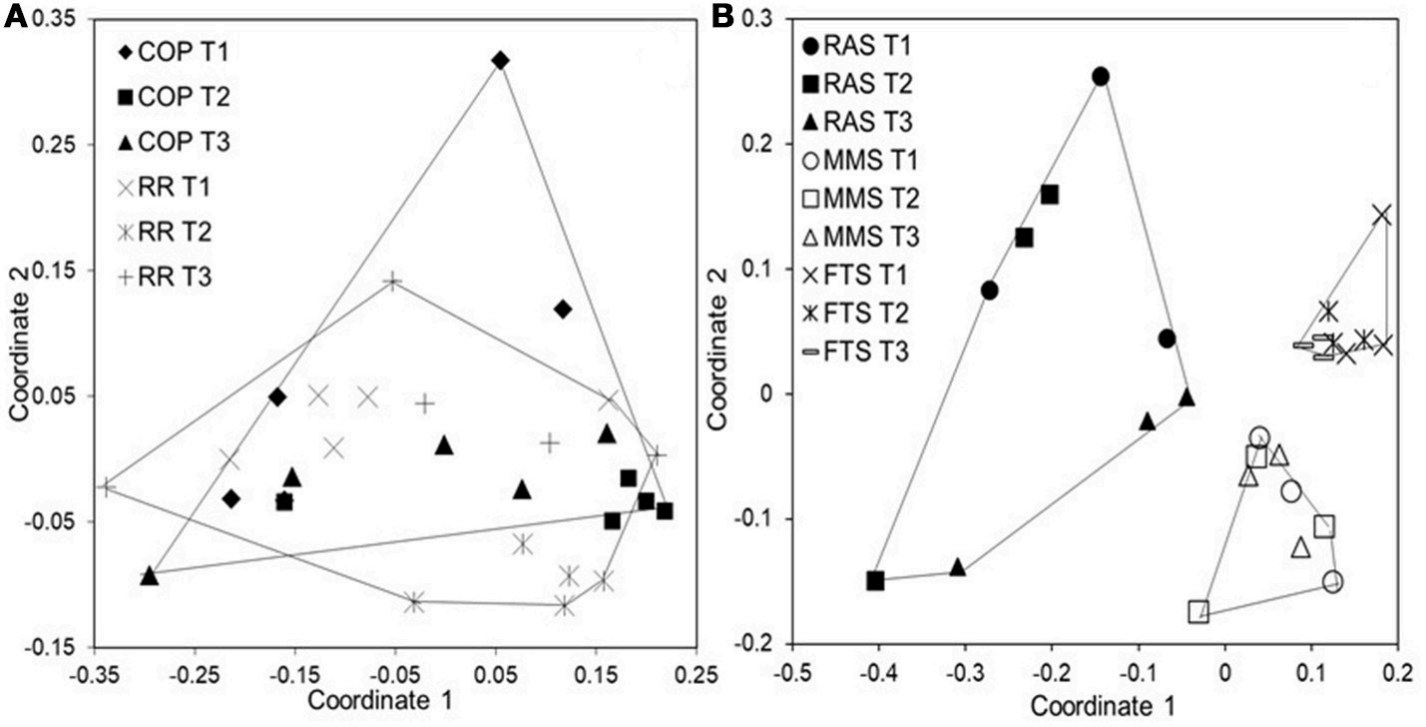
Non-metric MDS plots based on Bray-Curtis similarities for cod larval microbiota at 17 dph. **(A)** Microbiota from 30 larvae representing the diets COP (Copepods fed algae) and RR (rotifers fed algae); 5 individuals from each replicate rearing tank (based on data from Bakke et al., 2013). **(B)** Microbiota from 27 larvae representing the rearing water systems FTS, MMS, and RAS; 3 individuals from each replicate tank.

The community structure of the larval microbiota changed with age (Figure 5). At 8 dph the community was characterized by a high abundance of *Pseudomonas* (mainly one OTU), the presence of diverse β-proteobacteria (>13 genera), and a high abundance of *Bacilli*. At 17 and 32 dph the richness and diversity of the larval microbiota was low, and *Arcobacter* together with γ-proteobacteria (*Vibrio, Marinomonas*, and *Pseudomonas*) typically constituted more than 90% of the microbiota (Figure 6). At 61 dph the larval microbiota was more diverse again. The γ-proteobacteria included a variety of genera; mostly *Colwellia, Photobacterium, Leucothrix, Vibrio*, and *Pseudomonas*, and a high abundance of Rhodobacteraceae (α-proteobacteria). Many of the abundant genera in the microbiota of cod larvae have been identified in other marine fish larvae. For example, *Arcobacter* has been found in the microbiota of sea bass larvae (Lamari et al., 2013), *Vibrio* in haddock, Atlantic halibut and grouper (Griffiths et al., 2001; Jensen et al., 2004; Bjornsdottir et al., 2009; Sun et al., 2013), *Pseudomonas* and *Colwellia* in Atlantic halibut (Jensen et al., 2004), and *Marinomonas* in haddock (Griffiths et al., 2001).

The composition of the larval microbiota was highly different from the microbial communities in rearing water and associated with live food (Figure 5). The rearing water exhibited the highest diversity of all samples examined, and was typically dominated by diverse representatives of γ-proteobacteria (*Colwellia, Marinomonas, Vibrio, Pseudoalteronomas, Leucothrix*, and *Moritella)*, α-proteobacteria (Rhodobacteraceae) and different Flavobacteria. The bacterial communities associated with rotifer, copepod and *Artemia* cultures had some common features: i.e., the proportion of γ-proteobacteria was relatively low, whereas Rhodobacteraceae and Flavobacteria were relatively abundant. A high share of *Microbacterium* (approximately 40%) characterized the microbiota associated with the rotifer culture.

At present we cannot conclude on which microbiota that characterize healthy fish larvae. This is not a specific problem for larval rearing, but include most animals studied. There is at least two reasons for this. First, methodologically we do not have resolution at the species level. Second, conceptually it may be impossible to identify which species that are characteristic for healthy individuals. This is because it is not a species *per se*, but its activity that will decide ecosystem functioning. Many species may have the same functional role, and we know that healthy hosts have different species inventory. Thus, that the Anna Karenina principle that “Happy families are all alike; every unhappy family is unhappy in its own way.” (Tolstoy, 2002, p.1) likely does not hold for host microbiomes—there are many ways to happiness.

## Factors determining the composition of microbiota of larvae

Newly-hatched fish larvae live in close contact with the bacteria in their surroundings. When the mouth opens, the gastrointestinal (GI) tract is rapidly colonized, presumably by bacteria present in the surrounding water. However, bacteria associated with the egg, some coming from the mother, may also be involved (Hansen and Olafsen, 1999; Sullam et al., 2012). The growing larvae are continuously exposed to the various bacteria associated with the diet and the rearing water. In a rearing system, these environmental microbial communities are highly dynamic, and affected by e.g., excretion and feeding. Some of these bacteria will colonizing the larval gut and become a member of the intestinal microbiota. For fishes and terrestrial vertebrates, host genetics, phylogeny, trophic level, and diet are important factors structuring the gut microbiota (Ley et al., 2006; Rawls et al., 2006; Sullam et al., 2012). The factors contributing to the colonization at the individual level in developing fish larvae is not much studied, but has been assumed to reflect the environmental microbial communities, particularly those associated with the live food (Austin, 2006; Korsnes et al., 2006; Bjornsdottir et al., 2009; Nayak, 2010; Llewellyn et al., 2014). This assumption is probably too simplistic. Ecological processes like selection, dispersal and stochastic drift will likely influence the GI community structure (Dethlefsen et al., 2006; Robinson et al., 2010; De Schryver and Vadstein, 2014). Selection inside the host is determined by factors like diet, competition between microbes, host genetics, and developmental stage of the GI tract.

Among marine species, factors controlling the composition of the microbiota have been most thoroughly studied in cod larvae in two first feeding experiments. In one experiment, the effect of diet was investigated by rearing cod larvae with three different live food diets from 3 to 22 dph (Bakke et al., 2013): (1) copepods cultivated on the algae *Rhodomonas baltica* (COP), (2) rotifers cultivated with *R. baltica* (CR), and (3) rotifers cultivated with standard yeast/lipid (RR). From 18 dph onwards, all larvae were fed *Artemia*. In a second experiment, all rearing tanks received the same food, but three different rearing water systems gave different water microbiota: a flow-through system (FTS), a flow-through system with microbially matured water (MMS), and a recirculating system (RAS) (Attramadal et al., 2014). After 30 dph, all tanks received MMS water. Triplicate tanks were used for each regime in both experiments.

**Figure 7:**
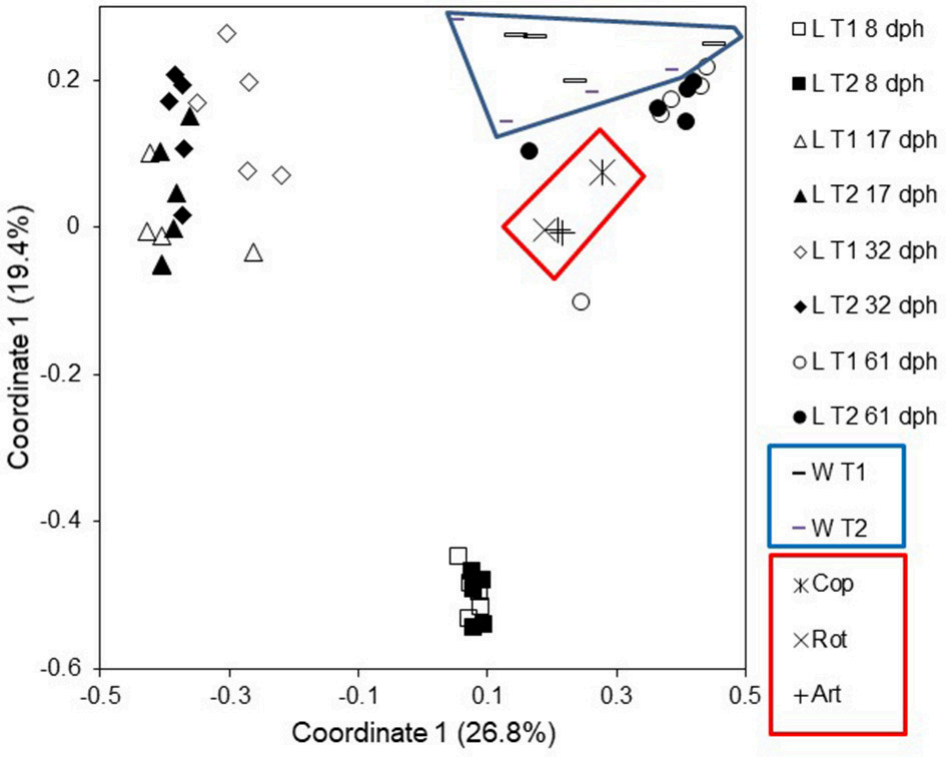
Principal coordinate analysis plot based on Bray–Curtis similarities for comparison of microbiota from larvae (L), rearing water (W), copepod (Cop), rotifer (Rot), and Artemia (Art) samples from Tank 1 (T1; fed copepods) and Tank 2 (T2; fed rotifers). Modified from Bakke et al. (2015). Reproduced with permission from John Wiley and Sons.

**Figure 8:**
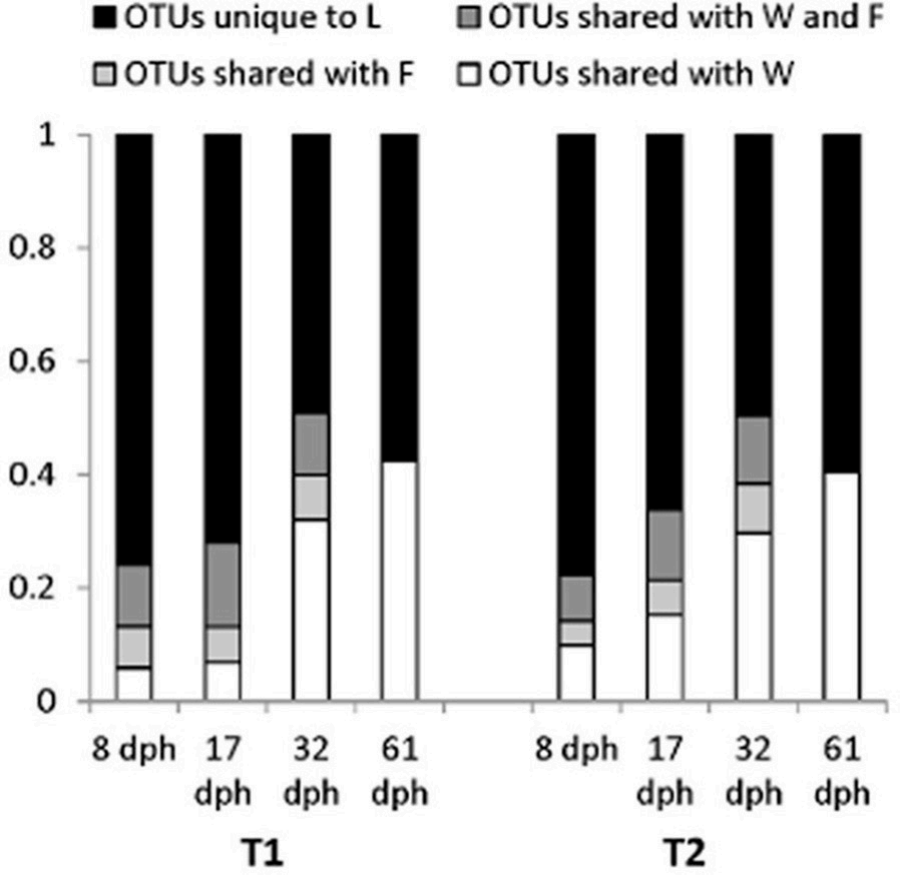
Bar graph indicating the fraction of larval microbiota Operational taxonomic units (OTUs) shared with concurrent water samples and relevant live food samples at each sampling time for Tank 1 (T1; fed copepods) and Tank 2 (T2; fed rotifers). L, larvae; F, food; W, water. OTUtaxonomic units represented by only one read (“singletons”) in the total dataset were omitted from the analysis. From Bakke et al. (2015). Reproduced with permission from John Wiley and Sons.

Surprisingly, the rearing water affected the larval microbiota more heavily than diet. Whereas different live food resulted in no difference in larval microbiota (Figure 6A; Bakke et al., 2013), larvae reared with different water systems had significant differences in their microbiota (Figure 6B, unpublished results). Several findings supported this conclusion. First, despite a transition in live food from rotifers or copepods to *Artemia* at 18 dph, the larval microbiota was similar at 17 and 32 dph. Second, after changing the rearing water system to MMS for all rearing tanks at 30 dph in the second experiment, the differences in larval microbiota between rearing systems disappeared. Third, larval microbiota was generally more similar to the water microbiota than to the live food microbiota, with respect to both community composition (Figure 7), and OUT inventory (Figure 8). A shift in the composition of the larval microbiota after onset of active feeding has been reported for several marine fish species, such as grouper (Sun et al., 2013), Atlantic cod (Brunvold et al., 2007; Reid et al., 2009), and Atlantic halibut (Jensen et al., 2004). However, most of these studies did not have a design suitable for addressing the importance of the food.

The above studies documented that among sources of environmental microbiota the water microbiota most strongly affect the microbiota of larvae. This may seem as a contradiction to the conclusion in the section on bacterial flows, and a clarification needs further research. The studies cited above does not give any insight into the reproducibility of the water microbiota at the system level. Giatsis et al. (2014) showed that inter-individual variation in the gut microbiota of tilapia within tanks was similar to the between tanks variation within the same RAS system. However, replication of the microbial community composition at the system level was not successful, and larvae grown in replicate RAS developed different gut microbial communities.

The cod larval microbiota study described above (Bakke et al., 2015), brought insight into the ontogeny of the microbiota. The community structure changed dramatically from 8 to 17 dph and from 32 to 61 dph, but was remarkably similar at 17 and 32 dph (Figures 5, 7). This temporal pattern could not be explained by stochastic processes (drift), and did not coincide with changes in the rearing conditions. Furthermore, the larval microbiota had very low similarity to the water and the live food microbiota, particularly at the first sampling (8 dph). Likely strong selection in the host structures the cod larval microbiota, and change in selection pressure due to development of the GI tract explain the observed temporal changes of the microbiota.

Some recent studies of other fish species support the conclusions above. In orange-spotted grouper larvae, a marine species not related to cod (Sun et al., 2013), the larval microbiota was more similar to water microbiota than to live food microbiota, and a shift in live food diet from oyster eggs to rotifers was not reflected in the larval microbiota. Contradicting results are found for zebrafish. Yan et al. (2012) concluded that the GI microbiota was deterministically assembled, but stochastic processes became more important in later developmental stages. Burns et al. (2016) found that the relative importance of non-neutral processes, such as microbe-microbe and host-microbe interactions, increased with age. However, their study did not include microbes in the environment.

When it comes to the effect of diet on fish larval microbiota, conclusions diverge. For sea bass larvae (Delcroix et al., 2014) and rainbow trout (Ingerslev et al., 2014) different feeding regimes resulted in significantly different larval GI microbiota. A possible explanation for the discrepancies could be that the formulated feed used in these experiments, resulted in larger variations in the composition of the diet and potentially higher selection pressure on the GI microbiota compared to different live food.

In conclusion, a number of factors, such as developmental stage, water microbiota, diet, and neutral processes like dispersal and drift, influence the microbiota associated with developing fish larvae. Of the sources of environmental microbes, the microbiota of the water most strongly affect larval microbiota. However, we need more studies to evaluate the relative importance of different factors influencing larval microbiota, and to understand the complex interactions between fish larvae and the bacterial communities of the environments.

## Eco-physiology of microbe-microbe and host-microbe interaction in fish larvae

**Figure 9:**
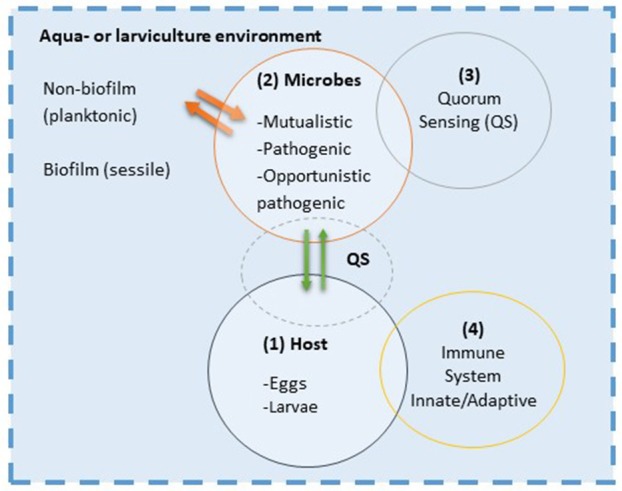
Host-microbe interaction concept model: In the aquaculture environment interaction between host (1) and microbes (2) basically start with a physical contact where bacteria attach to the surfaces of the host; the skin mucus, gills or gastrointestinal tract. Establishment of surface contact by microbes is through mechanisms such as chemotaxis and the use of fimbriae or pili (host cues might be utilized by microbe for that). Microbes communicate through chemical signaling, e.g., quorum sensing (QS; 3). This cell-to-cell signaling allows microbes to monitor the environment, and they alter cell population and/or activity in response to the chemical signal. There is also a cross-talk between quorum sensing and secondary messenger nucleotides, which are important for the fitness of the microbes by allowing switching between phenotypes in an unpredictable environment. This strategy also known as bet-hedging, is important for generating variable offspring in microbes and thus reduce the risk of being maladapted to the evolving (ontogeny) host environment. Changes from planktonic to sessile state (phase variation probably controlled by epigenetic mechanisms) in the biofilm and pili formation are example of bet-hedging strategy of bacteria. The host might sense the presence of colonizing microbes through their MAMPs (microbe-associated molecular patterns) modulating the innate immune responses (4) of the host. This in turn might shape the microbial community composition and its *in vivo* activity. The innate immunity plays a pivotal role in orchestrating the immune cells to determine the outcome of the host-microbial interaction which can be on the scale from mutualistic to pathogenic.

The stochastic and deterministic factors affecting the composition of the microbial community will create responses within the microbial community itself and bidirectionally between the larval host and its microbiota. *In vivo* identification and functional quantification of the microbial activities and host responses are of great interest. Few studies have focused on the bidirectional interaction in marine fish larvae, but some studies in mammals and plants have proven that communication mechanisms exist between prokaryote and eukaryote. Here, we propose a schematic diagram on the integration of several eco-physiological aspects that may play an important role in the interaction between fish larvae and their microbial populations (Figure 9). Many of the suggested interactions are so far hypothetical for fish larvae.

### Gnotobiotic systems to study fish-microbe interactions

Host-microbe interactions are of a complex and dynamic nature, and studying these interactions is a daunting task. For instance, many studies have been performed with probiotic strains or other types of microbial manipulations, but only few attempts have been made to clarify the exact beneficial effects microbiota have on their host beyond growth, feed conversion and survival. Interpretations of e.g., probiotic trials are often complicated due to the vast background microbiota present in the fish gut, water and food. To be confident of beneficial *in vivo* activity of a specific treatment, this “background noise” has to be removed. This can be achieved by the use of gnotobiotic systems, acknowledging that any finding under such experimental conditions would need validation under conventional conditions. Gnotobiotic animals are rendered free of all bacteria, and subsequently colonized with the strains of interest. This allows studies on the effects of specific bacteria on fish gene expression, metabolome and gut morphology. Gnotobiotic systems have been established for sea bass, Atlantic cod and tilapia (Dierckens et al., 2009; Forberg et al., 2011a; Situmorang et al., 2014) and the model species zebra fish (Kanther and Rawls, 2010) (full overview in Vestrum et al., 2018).

Many vertebrates show developmental anomalies when grown in the absence of microbiota. No such anomalies were observed in bacteria-free sea bass larvae during the first weeks of development, and bacteria-free sea bass and cod larvae showed high survival and no significant differences in growth compared to larvae with conventional microbiota (Rekecki et al., 2009, 2012; Forberg et al., 2011b). This difference compared to other vertebrates, can be due to the shorter experimental period for fish and detrimental larvae-microbiota interactions with the conventional microbiota. The effect of different probiotic candidate strains on gene expression of cod larvae revealed significant regulation of 14 “host response genes” involved in immune response, cell growth and nutrient uptake. Which bacterial species/strains that are present, and whether they are alive or dead makes a difference for how the different genes are expressed (Forberg et al., 2011b, 2012).

Gnotobiotic systems are an excellent tool to decipher the mechanisms underlying bacterial infection and disease. The gnotobiotic sea bass model was used for virulence assessment of different *Vibrio anguillarum* strains, visualization of colonization of the gut using GFP labeled *V. anguillarum*, and to investigate the role of sigma factor RpoS in *V.anguillarum* virulence (Rekecki et al., 2012; Frans et al., 2013; Li et al., 2014). Interestingly, using a set of 15 different *V. anguillarum* strains, no correlation was found between their genomic make-up and the virulence toward gnotobiotic sea bass larvae (Busschaert et al., 2015). In a followed-up with a larger collections of strains, Castillo et al. (2017) concluded that virulence of *Vibrio anguillarum* was linked to a set of chromosomal core genes in conjunction of pathogenic genomic islands, prophages and virulence factors, and a new set of gene clusters involved in biosynthesis, modification and transport of polysaccharides–all probably acquired through mobile genetic elements. Although quorum sensing has been demonstrated to be important in host-microbe interactions, using the *V. anguillarum* NB10 strain and isogenic mutants affected in several elements of the QS pathways, no effect could be documented on host survival in QS mutants (Milton, 2006) even under gnotobiotic conditions (Li, 2014). However, the RpoS mutant, a transcriptional regulator for stress related genes, was less virulent. This strain also upregulates indole, which might be a novel quorum sensing chemical (Li et al., 2014). This indicates the complexity of host-microbe interactions.

### Bacterial plasticity; phase variation and epigenetics

Colonization of larval surfaces or GI tract results in considerable changes in the environmental conditions of the microbes. Changes in e.g., oxygen, nitric oxide, light, and nutrient availability, modulate intracellular signals like cyclic AMP, cyclic di-GMP, cyclic GMP, and polyphosphate nucleotide. These signals allow bacteria to adapt to environmental changes, including regulation of virulence and biofilm formation (Srivastava and Waters, 2012; Kalia et al., 2013). The effect of cyclic di-GMP on biofilm formation and motility correlate with the expression of virulence factors, and regulate pathogenicity in several pathogens (*P. aeruginosa, Yersinia pestis, Vibrio cholera*, and *Salmonella enterica* serovar *thyphimurium;* Tamayo et al., 2010).

Gram-negative bacteria also respond to environmental changes by modifying the outer-membrane containing lipopolysaccharide, (LPS) (Bos and Tommassen, 2004). Different degree of acylation and glycosylation are some of the known processes that can change the surface of bacterial LPS (Boltaña et al., 2011). These changes in surface structure can result in antigenic and phase variation, and are a well-recognized adaptive strategy of the pathogen to survive and replicate in the microenvironment of their host. Bacterial phase variations are believed to be a random event that can be reversible and irreversible, occur at a high frequency (>10^−5^ per generation) and result in a phenotypically heterogeneous population (Henderson et al., 1999). An example of a phase variable system in fish pathogens is the production of bacterial siderophore for iron acquisition during infection events. Some pathogenic marine bacteria, e.g., *Vibrio anguillarum, Yersinia ruckeri*, and *Aeromonas salmonicida* subspecies *salmonicida*, are known for their production of siderophore to increase their virulence (Fernández et al., 2007; Lemos et al., 2009; Najimi, 2011). In *Vibrio harveyi*, phase variation also involve a switch from a luminous to a non-luminous strain. This shift coincided with an altered expression of putative virulence factors and modified virulence toward *Artemia fransciscana* under gnotobiotic conditions (Hong et al., 2016).

The adaptation and the phase variation of microbes during host-microbe interactions might find their origin in epigenetic modifications. The term epigenetics refer to stimuli-triggered changes in gene expression due to processes that arise independent of changes in the underlying DNA sequence (Gómez-Díaz et al., 2012). Different processes can influence changes in gene expression, but DNA methylation, histone modification and RNA mediated gene regulating are primary factors for epigenetic modification (Egger et al., 2004; Holoch and Moazer, 2015). These epigenetic mechanisms can control which genes that are expressed, and thus mediate eco-physiological responses when invading the host. In the dynamic host-pathogen environment many pathogens might develop the needed phenotype by epigenetic adaptation to the host's environment. Gómez-Díaz et al. (2012) have summarized the type of epigenetic mechanisms that have been shown to result in changes influencing gene expression related to virulence of pathogens.

### Bacterial-induced modification of host response and phenotype

Bacteria are no longer believed to exist as isolated single cell organisms (Kievit and De Iglewski, 2000). The discovery of cell-to-cell chemical signaling system (quorum sensing–QS) in order to communicate by signal molecules known as autoinducers (AI), was first discovered in the symbiotic relationship between the Hawaiian bobtail squid *Eupyrymna scolopes* and the bioluminescent *Vibrio fischeri* (Nealson et al., 1970). QS regulate e.g., mobility toward better environment or resources, production of virulence factor, and biofilm formation (Kievit and De Iglewski, 2000; Whitehead et al., 2001). QS uses several types of autoinducers with N-acyl homoserine lactone (AHL) as the major signaling molecules in Gram-negative bacteria (Schauder et al., 2001). The knowledge of these signaling molecules is remarkably vast. Most of the microorganisms producing AHL are known for their association with higher organisms in mutualistic or pathogenic relationships. Therefore, higher organisms have evolved mechanisms that can detect AHL signaling and respond to it (Whitehead et al., 2001).

Three examples are: First, evidence on the two-way signaling between human cells and quorum sensing molecules produced by bacteria was found in a study of cystic fibrosis infection by *Pseudomonas aeruginosa* (Shiner et al., 2005). The AHL molecules from *P. aeruginosa* freely diffuse into human cells, bind to intracellular immune signaling proteins, and facilitate the invasion of host target cell by the pathogen. Second, the symbiotic interaction between *E. scolopes* and *V. fischeri* has shown how the host responds to bacterial colonization by changing gene expression and protein production that leads to specific morphological developments in its light organ (Kimbell and McFall-Ngai, 2003). Third, in response to colonization by pathogenic bacteria or environmental changes, most animals produce stress hormones such as norepinephrine that regulate various aspects of the immune system (Verbrugghe et al., 2012). Production of stress hormones by the host also influenced pathogens such as *Vibrio parahaemolyticus*, resulting in increased cytotoxicity and enteropathogenicity via a type III secretion system (Nakano et al., 2007).

The immune system provides key responses in the interaction between host and pathogen. Recently, several reports on the immune system of teleosts highlight the role of innate immunity in first line defense before adaptive immunity reaches maturity. There is a considerable number of studies focusing on the innate immune system of fish, especially on Toll-like receptors signaling (Whyte, 2007; Rebl et al., 2010; Takano et al., 2010; see Boltaña et al., 2011). Fish larvae rely on their innate immune system in regulating danger signals responses against non-pathogenic or opportunistic pathogenic bacteria. The innate immune system recognize the presence of microbes by pattern recognition receptors such as Toll-like receptors (TLRs), through conserved microbe-associated molecular patterns. Continuous exposure to different microbes throughout the different life phases of e.g., fish larvae, requires orchestration of multiple TLRs signaling pathways dictating the status of interaction–mutualistic coexistence, asymptomatic infection or virulent disease (Brodsky and Medzhitov, 2009). It is likely that both mutualistic/commensal and pathogenic bacteria share conserved motifs. Yet, to invade the host, pathogenic bacteria need to gain a close access to the epithelial surfaces of the host. Moreover, pathogens secrete enzymes, toxins and other molecules which in turn generate danger signals in the host (Sansonetti, 2006), and activate the innate immune system. Several pathogens interfere with host immune responses through induction of epigenetic modifications, resulting in immunosuppression in the host (Gómez-Díaz et al., 2012). The complex interplay between mutualistic/commensal/pathogenic bacteria and the developing immune system of larvae is poorly understood, but could be used for microbial community manipulation strategies to improve larval viability.

### Conclusions

Research that clarifies mechanisms by which the microbiota promotes viability of larvae should have priority. The main issue is the phenotypic mechanism and its cause, but microbiota-larvae interaction should be studied at all levels from gene expression to the phenotypic response (Vestrum et al., 2018). Moreover, due to the complexity in these studies, we should benefit from the diversity of experimental systems available (Vestrum et al., 2018). As indicated above, the search for microbe-host interaction mechanisms is done with a wide variety of species, and this diversity provide inspiration and knowledge relevant for microbiota-larva interactions. Most studies focus on the effect of one or a few species, and not the holistic microbial community perspective advocated in this review. We see, however, a tendency to more community level consideration for e.g., plants (Lugtenberg and Kamilova, 2009) and corals (Peixoto et al., 2017).

## Steering and microbial management in larviculture

### General considerations

Marine juvenile production is characterized by large tank-to-tank variations in the performance of larvae. Increased reproducibility can be achieved by the use of antibiotics (Vadstein et al., 1993a; Munro et al., 1994; Skjermo et al., 1997; Verner-Jeffreys et al., 2004) or by methods to steer the microbial community of the rearing water (Vadstein et al., 1993a; Skjermo et al., 1997; Salvesen et al., 1999; Skjermo and Vadstein, 1999; Attramadal et al., 2012c, 2014). This indicates that the problems are related to detrimental fish-microbe interactions. The majority of the infections in marine fish larvae are believed to be caused by opportunistic microorganisms that become detrimental when the host's resistance is lowered by environmental stress (e.g., high bacterial load and physical disturbance) (Vadstein et al., 2004, in press).

The microbiota potentially influencing the larvae in intensive rearing systems includes the bacteria coming with eggs, live food, formulated feed, microalgae, water, and air, but water microbiota seems to have the strongest influence. The development of the microbial community depends on the composition of these sources as well as selection in the rearing system, including rearing tanks and fish gut. The best chance of succeeding in managing such complex systems probably lies in understanding and controlling the microbial community in each of these parts.

In 1993 Vadstein et al. proposed a strategy for microbial management in larval rearing with three different elements, and suggested several methods within each element (Table 3). Below we discuss the status of this strategy using Table 3 as a reference. In Table 4 we have listed some of the studies which have tested the methods.

**Table 3:** The three elements in microbial management in larviculture and examples of methods that can be used.

*Non-selective reduction of microbes*: • Disinfection of eggs and water (inc ozonation) • Reduction in input of organic matter • Removal of organic matter (*per se*, clay, biofilters, dilution, • Grazer control of bacterial biomass *Selective enhancement of microbes*: Composition and activity • Selection for desirable bacteria (inc Maturation, RAS, prebiotics) • Addition of selected bacteria to tanks (Probiotics) • Incorporation of selected bacteria in feed (Probiotics) *Improvement of resistance against microbes*: • Stimulation of general immune system • Modulation of general and specific maternal immunity

*Slightly modified from Vadstein et al. (1993a)*.

**Table 4:** Approaches to manage the microbial communities of each component part of the marine hatchery.

**Focus point**	**Aim**	**Action**	**References**
Eggs	Minimize bacterial growth on eggs	Surface disinfection	Salvesen and Vadstein, 1995; Salvesen et al., 1997
	Minimize transfer of harmful bacteria to tank	Microbial maturation of water	Skjermo et al., 1997; Salvesen et al., 1999
	Minimize transfer of organic matter to tank	High water exchange rates in incubator	
Intake water	Minimize transfer of harmful bacteria to tank	1. Disinfection, followed by	
	Minimize the chance of proliferation	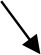 2. Regulation of microbial carrying capacity to resemble tank, followed by	Salvesen et al., 1999; Attramadal et al., 2012c
	of harmful bacteria in the tank	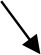 3. Microbial maturation	Skjermo et al., 1997
	Introduce neutral or beneficial bacteria	Addition of probiotic bacteria	Ringø and Vadstein, 1998; Makridis et al., 2000a
Particle addition	Minimize proliferation of harmful bacteria	Choosing the right species of algae for “green water”	Salvesen et al., 2000
		Choosing the right production regime for growing live algae	Salvesen et al., 2000
	Minimize transfer of organic matter to tank	Replacing algae with clay	Attramadal et al., 2012b
		Choosing the right type of clay	
Live feed	Minimize transfer of harmful bacteria to tank	Cleaning outside of live feed	
		▸ Disinfection	Munro et al., 1995
		▸ Cleaning with intake- or tap water	
	Introduce neutral or beneficial bacteria	Replacing gut flora of live feed	
		▸ With microalgae	Olsen et al., 2000
		▸ By addition of probiotic bacteria	Makridis et al., 2000b
		▸ By microbial maturation of water	Skjermo et al., 1997
Fish larvae	Improve larval resistance against infections	Improvement of immune system	Skjermo et al., 1997; Vadstein, 1997
		Optimizing and stabilizing physiochemical water quality	
		Optimizing welfare and minimizing negative stress	
		Optimizing nutrition	
Tank water	Minimize the proliferation of harmful bacteria	Continuous and efficient removal of waste	
		Reuse of water or recirculation with low level/without disinfection	Attramadal et al., 2012a,c
		Optimizing water exchange rates	
	Introduce neutral or beneficial bacteria	Addition of probiotic bacteria	Ringø and Vadstein, 1998; Makridis et al., 2000a

*Modified and extended from Attramadal (2011)*.

### Non-selective reduction of microbes

#### Disinfection

Disinfection is an efficient barrier against introduction of pathogens. Non-selective reduction of microbes can be achieved through disinfection of the surface of eggs (Salvesen and Vadstein, 1995; Salvesen et al., 1997), the outside of live food (Munro et al., 1995), and of water. Ozonation and UV-irradiation are popular methods to disinfect water, but have limited effects on bacteria associated with particles (Hess-Erga et al., 2008). Disinfection reduce the number of bacteria competing for substrate and the method thereby increase the access of organic matter per microbe. This leads to a temporal destabilization of the microbial community and favors proliferation of opportunistic r-strategists (Hess-Erga et al., 2010). Thus, disinfection of the water in the treatment circuit of a RAS may result in destabilization and regrowth of microbes in the rearing tanks (Attramadal et al., 2012a). Experiments with European lobster larvae (*Homarus gammarus*) in RAS with UV just before the rearing tanks gave a significantly different microbial community composition in the rearing tanks and a 20% reduction in the survival to stage IV compared to no treatment (Vadstein et al., in press). In conclusion, disinfection can be an efficient way of establishing a hygienic barrier, but it subsequently favor microbial regrowth of unwanted opportunistic microbes. These negative effects of disinfection can be counteracted by controlled recolonization of the water (Skjermo et al., 1997).

#### Reduction in input and increased removal of organic matter

The access of easily degradable organic matter determines the carrying capacity (CC) of microbes in the system. The CC is the maximum number of bacteria that can be sustained in the system over time. Hatching remnants, mortality, defecation, microalgae, and live food contributes to the organic load, and thus the CC (Figure 1). The place and method for reducing the loading of organic matter are of importance for the development of the microbial community. Rapid changes from low to high CC provide resources for opportunistic r-strategic species, and should therefore be avoided (Attramadal et al., 2016). The microbial growth in the rearing tanks should be reduced as much as possible to control the microbial community composition in the rearing tanks through the incoming water. A stable and relatively low CC throughout the rearing system, is probably the best way to avoid dominance of opportunistic microbes in the rearing water. A similar CC in the incoming water and tank will theoretically give few or no resources for growth in the rearing tank (Attramadal et al., 2016).

Direct removal of organic matter from rearing tanks may be achieved by manual or automated cleaning or by utilizing water current and tank design for self-cleaning. High dilution rates may be used to maintain a low CC in the rearing tanks, but simultaneously it increases the loss rate of the expensive live food. Clay can be used instead of microalgae for contrast in the rearing tanks, and will reduce the supply and increase the removal of organic matter (Attramadal et al., 2012b). Most of the particles in hatchery rearing waters are small and hard to remove with filtration. Heterotrophic microorganisms in the biofilter of RAS consume organic matter, but they also consume oxygen and produce CO_2_ and ammonium, which reduces the water quality (Blancheton, 2000; Sharrer et al., 2005). Water treatment methods for continuous harvest of small particles and microbes from the water, like protein skimmers, sand filters and membrane filtration is probably the best options for maintaining low levels of organic matter in hatchery RAS. In conclusion, a stable and low microbial CC should be targeted throughout the system, and large differences in CC between incoming water and rearing water should be avoided to minimize growth of opportunistic microbes.

#### Grazing/predation

Predation is a major mortality factor for bacteria in most ecosystems. In most aquatic systems protozoa are the main predators on bacteria, but some metazoan can be important. Both rotifers and *Artemia* are able to feed on bacteria, but with clearance rates that are typically only 5–20% of the maximum rates (Vadstein et al., 1993b; Makridis and Vadstein, 1999). However, because the live food is present at high densities in the first feeding tanks the population impose a significant mortality rate on bacteria. Two and four days old *Artemia* impose a bacterial mortality rate of 0.36 to 1.20 day^−1^ at population densities of 1000 per L. For rotifers at densities of 30,00–50,00 per L, the rate is lower (0.1 day^−1^). No published studies deal explicitly with implications of predation as a non-selective method for reduction of bacteria in aquaculture, but indirect evidence is found in e.g., Thompson et al. (1999).

The use of bacteriophages to induce mortality on bacteria were not mentioned in Vadstein et al. (1993a), but this 100 years old method needs some attention. First, phages are selective, even to the strain level. This makes phages suitable for selective reduction of specific problem bacteria. Second, bacteria have evolved antiviral defense systems, and thus there is a potential resistance problem with their use. How fast resistance is developed is still under debate, with contradicting results from laboratory studies and clinical trials. It is claimed that resistance to phage infections develops much slower than antibiotic resistance, but may still limit the usefulness of phage therapy (Ormälä and Jalasvuori, 2013). Moreover, studies with *V. anguillarum* indicated highly variable phage protection mechanisms in two closely related strains, emphasizing the challenge of using phages to control vibriosis in aquaculture (Tan et al., 2015). For a recent review on phage therapy see Doss et al. (2017).

The potential of increasing mortality of bacteria by the use of predators and phages is hardly studied in an aquaculture setting. In future work it is important to consider the differences in selectivity and resistance with the two methods, and to consider the potential for regrowth by bacteria.

### Selective enhancement of microbes

We lack knowledge on which microbes that benefit the larvae. This is a general problem for health care of animals—including humans. Moreover, it is likely not the species, but their activity that is critical, and this functionality can be achieved with different species inventory. (see sections Community composition of microbiota associated with marine larvae and Eco-physiology of microbe-microbe and host-microbe interaction in fish larvae). As an alternative way around this dual problem, it has been hypothesized that larvae reared in water dominated by K-strategists (mature microbial communities) will perform better, because these larvae are less likely to encounter opportunistic (r-selected) microbes and experience detrimental host-microbe interactions (Vadstein et al., 1993a, in press; Skjermo et al., 1997). To improve the microbial environment of the larvae, the fraction of opportunistic bacteria, including potential pathogens, should be reduced and the general diversity of bacteria, including mutualistic species and K-strategists, should be increased.

Selective enhancement of bacteria in intensive rearing of larvae has used two different approaches:

1. *K-selection*: directional selection to increase the microbial diversity and stability, and to reduce the fraction of potentially harmful bacteria (Skjermo et al., 1997; Salvesen et al., 1999; Attramadal et al., 2012a,c, 2014).
2. *Probiotics*: the addition of beneficial bacteria to water or feed (Gatesoupe, 1999; Makridis et al., 2000a; Skjermo et al., 2015).

Directional, community level selection is a general Darwinian approach, and normally done with a continuous selection pressure and without assumptions on the identity of species selected for. The probiotic strategy is a perturbation that requires knowledge about the species that are beneficial. Per definition, the addition of probiotics reduces diversity in the sense of evenness, whereas K-selection increases diversity. It is a challenge to make the probiotics remain in the systems (Skjermo et al., 2015), whereas directional selection favors a stable microbial community (Vadstein et al., 2004; Attramadal et al., 2014). The addition of probiotics in combination with a substrate selecting for the probiont(s) added (i.e., synbiotic approach), was not mentioned in Vadstein et al. (1993a) and is a little explored strategy.

#### Selection for desirable bacteria

K-selection can be set up by securing competition for resources, i.e., low nutrient supply per microbe (Vadstein et al., 1993a, in press). This can be obtained by two technologies. First, microbial maturation of water (MMS) can be done by controlled recolonization of the disinfected intake water by passing it through a heterotrophic biofilter occupied by K-strategists (Skjermo et al., 1997). MMS has been shown to increase viability of marine fish larvae, including appetite, growth, survival and stress tolerance (Skjermo et al., 1997; Salvesen et al., 1999; Attramadal et al., 2014; reviewed in Vadstein et al., in press). MMS is stable and robust in terms of bacterial numbers and composition, and operates close to the CC of the incoming water. However, the rearing tanks have a significantly higher CC than the intake water due to feeding (Attramadal et al., 2014). K-selection at the CC of the rearing tanks hypothetically give higher microbial stability and limit the growth of bacteria in the water directly in contact with the fish. To increase the CC of MMS the water going into the rearing tanks can be added organic matter to the maturing unit (biofilter). Comparisons between two MMS with low or elevated (F-MMS) microbial CC in incoming water to rearing tanks supported the hypothesis, and showed reduced regrowth of bacteria and a more stable microbial community composition in F-MMS (Attramadal et al., 2016).

Another solution to mature water is to reuse the wastewater to feed the heterotrophs in the biofilter in RAS. RAS secures a similar CC throughout the system, and an extended time for maturation because of the high hydraulic retention time (days). Provided limited use of disinfection, the microbial growth potential is low throughout the system as most organic matter is consumed in the biofilter, and thus the risk of invasion by new bacteria is low (Attramadal et al., 2012a). Even though the microbiota of biofilm and water differ significantly (Bakke et al., 2017), a matured biofilter is likely a stable source of K-selected microbes to the water (Blancheton et al., 2013). However, the interaction between bacteria in biofilter and water is poorly understood.

In a study by Attramadal et al. (2014), the microbiota of the rearing water was significantly different between treatments, and the survival of Atlantic cod larvae at 32 days post hatching was more than 60% higher in RAS (29 ± 3%) and MMS (28 ± 5%), than in the traditional flow through system (17 ± 4%). This together with previous studies (Skjermo et al., 1997; Salvesen et al., 1999; Attramadal et al., 2012a,c) supports the hypothesis that RAS and MMS promotes K-selection with beneficial effects on the fish. Attramadal et al. (2014) supports the prediction regarding CC in RAS compared to FTS and MMS, and showed a more stable composition of microbes over time in the rearing water in RAS compared to the other two systems. The drawback of RAS is the accumulation of waste products in the water. However, efficient water treatment technologies are established, and the low fish biomass results in low waste production during the live food period.

#### Addition of probiotics through rearing water and feed

Probiotics were originally defined as feed supplement (Fuller, 1989). Research on probiotics in aquaculture started in the late 80 s (see Gatesoupe, 1999), and it is the most studied microbial management method in aquaculture. The microbial strains are mainly selected based on their ability to produce antimicrobial compounds (antagonism), but also other characteristics (Vine et al., 2006). There are strict regulations for adding biological agents to commercial feed in Europe (EFSA Panel on Biological Hazards, 2012), but addition to the water has less strict regulations. The topic is reviewed extensively during the last 25 years. Documented positive effects are divers (Tovar-Ramirez et al., 2004, 2010; Lamari et al., 2013) but mode of action is generally not known. Despite ontogenetic differences between fish and mammals, some mechanisms can be common, e.g., stimulation of gut maturation by yeast polyamines (Buts et al., 1994). As stated above (5.4) more research should be directed toward studying mechanisms of action. Moreover, positive effects with probiotics may mainly reflect the suboptimal conditions for fish under traditional rearing conditions.

Protection against opportunistic pathogens is an important and desirable mode of action, and the search for probiotic candidates with such properties has been going on for several decades. Many different mechanisms can make bacteria antagonistic to pathogens. One other focus has been to find candidates that become part of the normal gastrointestinal microbiota of the developing fish larvae, and thus reduce the possibilities for pathogens to adhere and infect the larval intestine (De Schryver and Vadstein, 2014). Candidates suitable for the complete larval stage probably do not exist due to the changing selection pressure in developing larvae (Bakke et al., 2015).

Probiotic bacteria can be introduced to the fish larvae by addition directly to the rearing water (Reitan et al., 1998) or by bio-encapsulation in the live food (Makridis et al., 2000a). Probiotic bacteria must persist long enough in the intestine to have a beneficial effect against opportunistic pathogenic bacteria, and the ability to adhere to and colonize mucosal surfaces are important properties. In a study with cod larvae the time window for successful colonization by four candidate probiotic bacteria from cod larvae (Fjellheim et al., 2010) was investigated. The strains were added at seven stages from hatching until 45 dph (Skjermo et al., 2015). Despite the origin of these probiotic candidates and their confirmed ability to colonize and grow in mucus, only one strain was able to colonize at significant densities. The colonization was only successful the first week after hatching and after weaning, and close to the detection limit in between. Independent of larval age, the colonization did not persist for more than 3–4 days for any of the candidates, indicating poor competitive ability in the larvae. The result confirms that the gut microbiota of fish larvae is transient and change rapidly with development (Olafsen, 2001; Bakke et al., 2013, 2015), and long-term steering of the composition of the microbiota is difficult by a single perturbation. The durability of probiotic bacteria will thus depend on a continuous supply, or a selection regime that improves the persistence by e.g., supply of a prebiotic (see below).

Further research should focus on the effect on larvae of the continuous supply of probiotic strains to the water or the live food organisms. Colonization of biofilters or tank walls with probiotic candidates could be a practical solution for a continuous supply of probiotics into the rearing water. Alternatively, probiotics could be grown on a carbon source available in a hatchery, such as the carbon sources released by hatching *Artemia* and used to continuously inoculate larval tanks. This concept has only been explored at the experimental level (Dang et al., 2009). Moreover, the consequences of probiotics for reducing diversity and evenness of the microbial community needs attention (De Schryver and Vadstein, 2014). Finally, it is important that aquaculture benefit from concepts and knowledge developed with other microbiota-host systems (e.g., Lugtenberg and Kamilova, 2009; Singh et al., 2013;Peixoto et al., 2017).

#### Prebiotics

Prebiotics are non-digestible food ingredient that promotes the growth of beneficial microorganisms in the intestine. They may be a tool for selection of beneficial bacteria in the host. As for probiotics, the concept of prebiotics was initially developed for humans. Prebiotics used in humans have also been tested in aquaculture (Ringø et al., 2010). However, these prebiotics selects mainly for lactic acid bacteria, which are not a dominant member in the gut microbiota of fish and may thus not be appropriate. Still, many of the marine pathogens primarily use proteins/amino acids as carbon source, and oligo- and polysaccharides may therefore promote growth of non-pathogenic species.

There are several reports on positive effects of oligosaccharides on growth of several fish species (reviewed by Ringø et al., 2010), but few studies have been done on larvae. Mannanoligosaccharides, supplied by enrichment of live food, improved survival of cobia larvae after exposure to stress conditions (Salze et al., 2008), and they improved growth and survival of Nile tilapia larvae after challenge with *Streptococcus agalactiae* (Samrongpan et al., 2008). None of these studies determined the effect on the intestinal microbiota, and it cannot be ruled out that the effects are immunological. One complicating challenge is that pathogens are found in the same or closely related genera as potential probiotics (e.g., *Vibrio, Marinomonas*), and a selective enrichment of naturally occurring probiotic candidate may also result in selection for pathogens as competitive ability seems to be related to phylogeny.

A more targeted strategy than sole use of prebiotics to select for naturally occurring beneficial bacteria, is to improve the persistence of added probiotics—the so-called synbiotic concept. In this way, it is possible to omit or reduce the need for a continuous supply of probiotic bacteria. In order to explore this strategy, growth on potential selective substrates should be one of the selection criteria when screening for probiotics. In a further characterization of the four probiotic candidates for cod larvae identified by Fjellheim et al. (2010), growth on marine polysaccharides and recognized dietary fibers was evaluated. One of the candidates had high growth rates on barley β-glucans and laminaran, a β-glucan from seaweed (unpublished). Supply of these substrates may therefore improve the poor persistence of probionts observed by Skjermo et al. (2015).

The pre- and symbiotic concepts need more attention as microbial management methods in larviculture. However, the research should be based on an analysis of differences between mammalian and teleost microbiota, and it should have more focus on the microbial community and its activity with the goal to better understand the cascade of prebiotics on selection and activity of the microbiota, and further effects on viability of the fish. Thus, we should abandoned the empirical approach dominating so far.

### Improvement of resistance against microbes

The ability of the fish to resist potentially harmful bacteria has received some interest, but has mainly focused on various components of the immune system (Vadstein, 1997). Stress influences the immune system of animals, and stressed animals are more susceptible to infections. For specific immunity, larvae rely on maternal immunoglobulins, as the specific immunity is not fully developed until after metamorphosis (Vadstein, 1997; Rombout et al., 2011; Vadstein et al., 2013). It is possible to manipulate antibody composition and level in eggs and larvae through immunization of the mother, but too little is known to fully evaluate the potential of this method. The innate immune system is the major defense against microorganisms in fish larvae. This is due to a non-functioning specific immune system, but also because specific pathogens do not seem to be the main reason for the mortality during the larval stage. Although a large number of immune system constituents known from mammals also have been described for fish, relatively little is known about larval stages of marine fish (Vadstein, 1997; Rombout et al., 2011; Vadstein et al., 2013).

Several studies with different immunostimulants have demonstrated that administration of immunostimulants to larvae has a beneficial effect on survival, growth and resistance to pathogens (Vadstein, 1997; Bricknell and Dalmo, 2005). Moreover, live food can be used as a vector for immunostimulants (Skjermo et al., 1995). Despite the fact that the knowledge on fish immunology has developed rapidly the last decade, the progress within the field of immunostimulation as a microbial management method, has developed slowly during the same period. Studies that target stimulation of the immune system in connection with perturbations/stress and accompanying immune suppression, are few for larval stages of fish. Such studies are required to evaluate fully the potential of immunostimulation.

## Conclusions and recommendations

Major new findings highlighted above are: (1) The importance of the rearing regime for the flow of bacteria that larvae are exposed to in the rearing tank. The number of bacteria in incoming water, the hydraulic retention time and the growth of bacteria in the rearing tank due to production of resources for growth are three factors of main concern. (2) The microbiota of larvae is much more diverse than indicated by cultivation-based methods, and it changes through larval development. A number of factors influence the microbiota associated with developing fish larvae. Selection within the host is strong, and the environmental microbes most strongly affecting the microbiota of larvae is the water microbiota. (3) Microbes have an eco-physiological plasticity, resulting in dynamic microbiota-host interactions. The effects of the microbiota on the host are poorly studied in general, but more so in fish. However, the microbiota affect a wide variety of functions and they are essential for normal development of the host. (4) A multitude of methods for microbial management in larval rearing have been proposed, and some of them are promising and could be implemented in the aquaculture industry. However, the diversity is low in research on microbial management methods.

Along with the concept developed in this review, general recommendations can be made for future research. We have identified three priority areas, and we give some specific examples for further research.

The first area of research is related to the quantification and the control of the amount of microbes reaching the larvae. Focus need to be on the interaction between rearing technology and microbial ecology. Two aspects that need particular attention are hydraulic retention time in rearing tanks, and growth of microbes in the tanks and the significance of differences in carrying capacity of inflowing water and tank water.

The second area of research is related to the control of the type of microorganisms in larval tanks. Community level K-selection of microbes by water treatment in aquaculture systems is a promising strategy that still needs further documentation. Attention should be given to systems design and the capacity for K-selection, and on the development of the microbial community composition under K-selection. The gut microbiota of marine fish larvae has a dynamic and transient nature. A strategy for the selection and the introduction of probiotic bacteria to larvae should ensure continuous supply. Special attention should be given to the live food period, as this seems to be the most critical phase and it is driven by microbial problems.

The third area of research is related to how mutualistic host-microbe interactions are established, and the metabolic and developmental impact of microbes on larvae. This relates to the unexplored field of microbial activity of resident and transient microbes in the larval gut. It seems as microbes develop a different gene expression pattern inside the gut, but it is also possible that life inside the gut requires a change in the phenotypic status of microbes. This could be modulated by epigenetic control. Microorganisms will have an influence on the larval metabolism and development. Research in this field has been hampered by the stochastic colonization of the gut, making it difficult to reproduce experiments. In the future a systematic investigation of the immediate and sustained effects of incoming or locally produced microbe-associated molecular patterns (MAMPs) is a necessity. The gnotobiotic systems available for several species provide full microbial control and thus better reproducibility when it comes to studies on host-microbe interactions. Immunostimulation seems to be a method with potential, but requires further documentation on the effects of larval metabolism and investigations of relevant use. Production scale verification will also be required.

Better insight into point 1 and 2 will give the fish farmer tools to gain microbial control. Some microbial management tools can be applied already today, although not necessarily in an optimal way. Point 3 will improve significantly the general knowledge base, and serve as a reservoir for future knowledge-based development of rearing conditions that increase the economic, environmental and social sustainability of aquaculture.

Further studies on microbial management strategies should diversify methodologically and experimentally-preferentially in combination. This area has received considerable attention during the last two decades, but it has not fully explored the potential presented here (Tables 3, 4). A relatively high fraction of studies has considered disinfection and probiotics. Some of these studies are promising, but there is a tendency of system specificity that make generalizations difficult. Moreover, for e.g., studies on probiotics the studied effect variables are relatively crude (mainly survival and growth of fish), and give no insight into mechanisms of action. Due to the impact of early life for later performance and the significance of epigenetics, further studies on microbial management should also consider the impact of the treatments beyond metamorphosis.

It is time for a paradigm shift regarding our understanding of host-microbe interactions in general, and more specifically in aquaculture. Research published recent years have shown that mutualistic interactions between host and microbiota is the normal. However, stress induced by e.g., intensive cultivation, can compromise this mutualism. Moreover, the interactions between microbes and a healthy host involves a multitude of mechanisms not accounted for by a single population/species. Thus, different from pathogenesis, the study of healthy individuals require consideration of the microbiota at the community level. In both research and industry we should counteract the probability of dysbiosis under a paradigm were we consider most members of the microbial community as positive actors for a more sustainable aquaculture, and we should search for knowledge and methods for microbial community management.

## Author contributions

PB and OV outlined the paper, and major contributions to the text for specific sections was written by OV, KA, YO, IB, and PB. The other authors provided minor input of text. All authors contributed with general comments to the overall manuscript.

### Conflict of interest statement

The authors declare that the research was conducted in the absence of any commercial or financial relationships that could be construed as a potential conflict of interest.
